# Pharmacist-led interventions in optimising the use of oral anticoagulants in patients with atrial fibrillation in general practice in England: a retrospective observational study

**DOI:** 10.3399/BJGPO.2023.0113

**Published:** 2024-04-17

**Authors:** Raman Sharma, Syed Shahzad Hasan, Ishtiaq A Gilkar, Waheed F Hussain, Barbara R Conway, Muhammad Usman Ghori

**Affiliations:** 1 Department of Pharmacy, School of Applied Sciences, University of Huddersfield, Huddersfield, UK; 2 Little Horton Lane Medical Centre, Bradford, UK; 3 Clarendon Medical Centre, Bradford, UK

**Keywords:** atrial fibrillation, general practice, anticoagulation, direct oral anticoagulants, pharmacists, prescribing

## Abstract

**Background:**

Oral anticoagulation (OAC) is the mainstay of treatment for the prevention of strokes in patients with atrial fibrillation (AF). Direct oral anticoagulants (DOACs) account for increasing OAC in patients with AF. However, prescribing DOACs for patients with established AF poses various challenges and general practice pharmacists may have an important role in supporting their management.

**Aim:**

To investigate the effectiveness of pharmacist-led interventions in general practice in optimising the use of OAC therapies in AF.

**Design & setting:**

A retrospective observational study in general practices in Bradford.

**Method:**

The data were collected retrospectively from 1 November 2018–31 December 2019 using electronic health record data. The data were analysed: 1) to identify patients with AF not on OAC; 2) to describe inappropriate DOAC prescriptions; and 3) to calculate HAS-BLED scores.

**Results:**

Overall, 76.3% (*n* = 470) of patients with AF received OAC therapy, and of these, 63.4% received DOACs. Pharmacist-led interventions increased DOAC prescribing by 6.0% (*P =* 0.03). Inappropriate DOAC use was identified in 24.5% of patients with AF, with underdosed and overdosed identified in 9.7% and 14.8%, respectively. Post-intervention, inappropriate prescribing was reduced to 1.7%. The mean HAS-BLED score decreased from 3.00 to 2.22 (*P*<0.01). Successful transition from vitamin K antagonist (VKA) therapy to DOACs was achieved in 25.7% of patients.

**Conclusion:**

Pharmacist-led interventions have successfully improved the use of OAC therapies in patients with AF, and effectively managed the bleeding risks and transition from VKA to DOAC therapy, in line with guidelines.

## How this fits in

Given the relatively recent implementation of pharmacists in patient-facing roles within general practice, there is a scarcity of published research in this domain, particularly concerning the assessment of pharmacist-led interventions in chronic conditions such as atrial fibrillation (AF). This research aids in assessing the role of GP pharmacist-led interventions in optimising oral anticoagulants in patients with AF in general practices in England. The findings of this research will enhance the understanding of anticoagulant optimisation within primary care, providing valuable insights for healthcare professionals.

## Introduction

Atrial fibrillation (AF) is the most common sustained cardiac arrhythmia in adults worldwide, with an increased risk of stroke.^1^ AF prevalence in adults is estimated to be between 2% and 4%, with a two-to-three-fold increase expected in the general population over the next decade.^2,3^ UK prevalence of AF has increased in all age categories and sexes, with higher prevalence in males and the most significant increase in patients aged >85 years.^4^ Public Health England estimates a prevalence of 2.5% in the population.^5^

Oral anticoagulation (OAC) remains the mainstay of treatment for stroke prevention, recommended by the National Institute for Health and Care Excellence (NICE).^6^ Current guidelines recommend OAC for all patients with AF and CHA_2_DS_2_VASc score of ≥2, and in males with a CHA_2_DS_2_VASc score of 1, taking bleeding risk into consideration.^1,7^ OAC options include the vitamin K antagonist (VKA), warfarin, and direct oral anticoagulants (DOACs), which include apixaban, dabigatran, edoxaban, and rivaroxaban. The prescribing trend for DOACs has shown an overall increase, accounting for 62% of all OACs in 2019 compared with 16% in 2015.^8^ Randomised controlled trials (RCTs) have demonstrated that DOACs are comparable with warfarin in stroke prevention.^9–12^ Owing to their efficacy in clinical practice,^13,14^ DOACs are now considered the first-line treatment.^1,7^ Significant variations remain within geographical regions in England regarding DOAC uptake, with rates of 130 per 1000 patient population in Greater London and 232 per 1000 patient population in Yorkshire and Humber in 2019.^8^ It has been shown that local clinical guidance increases DOAC usage when listed as first-line treatments,^15^ with edoxaban recently identified as the preferred DOAC.^16^ At the time of writing, the effect of this on DOAC usage in England is yet to be quantified.

Major bleeding risks persist with OAC use but DOACs show a favourable net clinical benefit compared with warfarin.^17^ Annual rates of major bleeding with VKA therapy in AF range from 1.4%–3.4%,^18^ with severe bleeds, such as intracranial haemorrhage (ICH), occurring in 0.1%–2.5% of patients.^19^ The different classification of bleeds with OAC are described in Supplementary Table S1. DOACs exhibit lower incidence of major bleeds (–14%) and ICH (–52%) compared with VKA.^13,18^ Bleeding risk factors, influenced by age, comorbidities, and concurrent medications, should be periodically addressed.^20^ The HAS-BLED score was developed, validated, and recommended for bleed risk assessment by NICE,^6^ but it was superseded by ORBIT in 2021,^7^ with the scoring criteria for each described in Supplementary Table S2.

Inadequate stroke prevention treatment for AF has been widely reported, burdening patients, carers, and healthcare systems.^21–23^ Moreover, the number of GPs in England declined by 5% between 2015 and 2021 despite a 4% population increase and more complex health requirements posing a further challenge to the adequate treatment options for patients with stroke.^24^ National efforts have prioritised AF detection, OAC initiation, and optimisation,^25–28^ but OACs remain underutilised in about one-third of eligible patients.^29–31^ Furthermore, clinicians' individual perceptions pose barriers to prescribing, including medication safety concerns, polypharmacy issues, limited understanding and experience in AF management, and perceived risks of falls and bleeding.^32^ Clinical decision making appears to be based on individual preference rather than a systematic approach by different clinicians.^33^ Despite these data, efforts to improve AF management in general practice are not extensively documented. Pharmacist-led care has been shown to improve screening rates in general practice,^34–36^ and improve patient understanding of and outcomes associated with OAC therapy in AF management. A secondary care-based specialist pharmacist supported the practice-based pharmacist (PBP) workforce with AF management, reduced bleed risk by deprescribing antiplatelet therapy, and guideline adherence.^37–39^

The current study aimed to investigate the effectiveness of pharmacist-led interventions in optimising the use of OAC therapies in patients with AF in general practice settings in England.

## Method

### Study design and setting

A multi-centre retrospective observational (pre-post) study was undertaken across general practices in the City Health Bradford GP Federation from 1 November 2018–31 December 2019. It compared the prescribing practices for OAC treatment (pre-intervention) to outcomes for patients managed by pharmacist-led medicines optimisation interventions in AF (post-intervention).

### Study population

The study population consisted of patients aged ≥18 years with a confirmed AF diagnosis and on the Quality and Outcomes Framework (QOF) AF register.^40^ Patients registered at one of the City Health Bradford GP Federation member general practices with a confirmed diagnosis of AF were included with no limitations placed on sex and ethnic group. However, patients with a confirmed diagnosis of valvular AF were excluded from the study, owing to their OAC treatment modalities varying from those patients diagnosed with non-valvular AF.

### Outcome measure

The primary objective was to determine OAC prescribing pre- and post-intervention by pharmacists. A summary of intervention details related to different phases of the current project has been tabulated in Supplementary Table S3. Secondary outcomes included determining the proportion and classification of inappropriately prescribed OAC treatments and bleed risk, and consequent intervention analyses related to these parameters.

### Risk stratification and OAC treatment

Baseline characteristics, including age, sex, previous stroke history, and any hospital admissions in relation to thrombolytic events, were collected via the medical record. Blood biochemical measurements of estimated creatinine clearance (CrCl) were calculated using the Cockcroft–Gault calculator in the clinical system, SystmOne, using actual body weight as recommended for DOAC dosing.^41^

The CHA_2_DS_2_VASc score was used to evaluate the risk of stroke in patients with AF.^42^ HAS-BLED score is a composite of modifiable and non-modifiable bleeding risk factors.^42^ The score can be stratified as low (0 points), intermediate (1–2 points), and high risk (≥3 points).^43^ Where data for time in therapeutic range (TTR) were not available for patients on VKA therapy, 0 points were allocated when undertaking bleed risk assessment.

**Figure 1.**
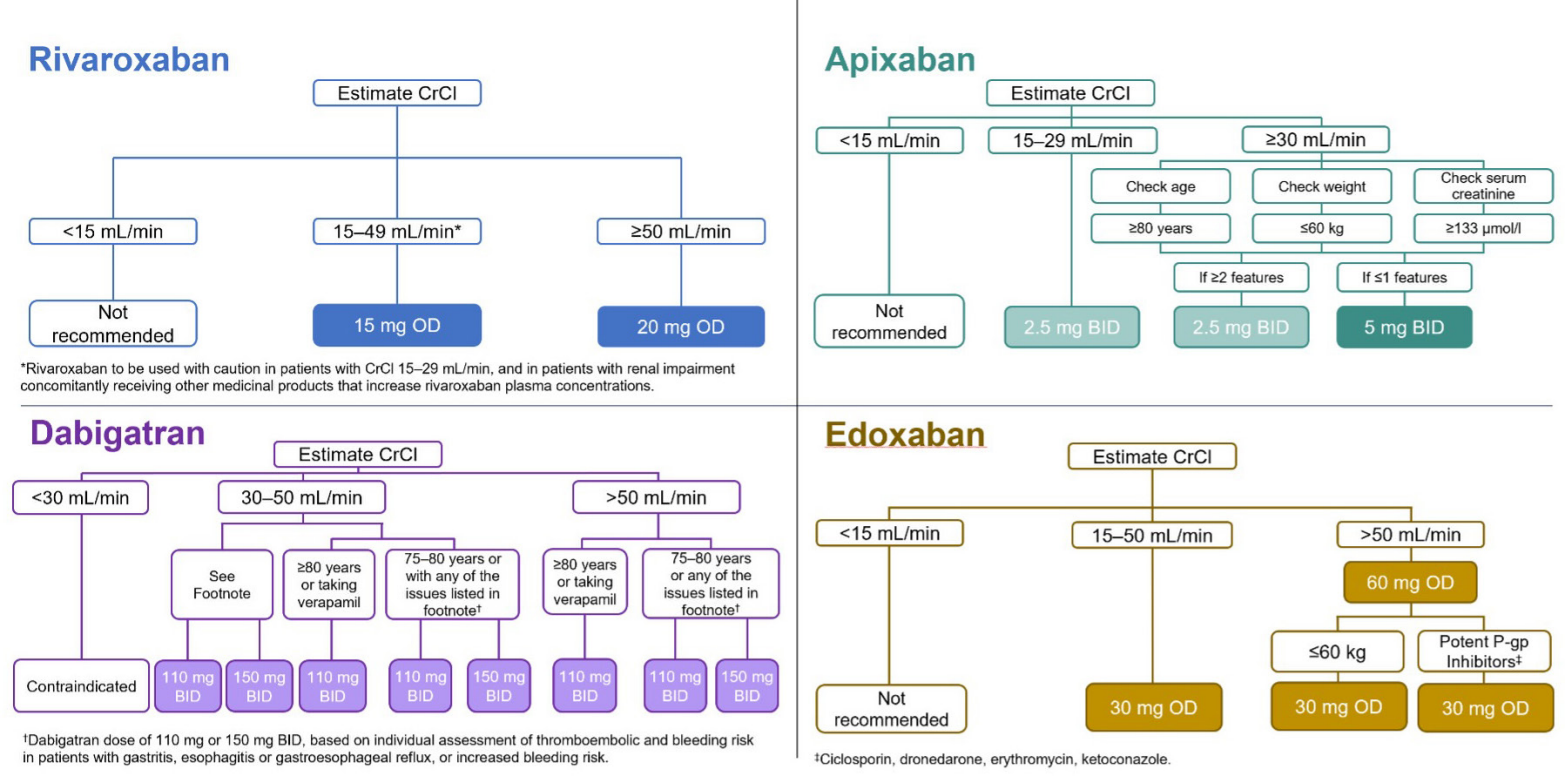
Direct oral anticoagulants dosing algorithms.^44–49^ BID = twice daily. CrCl = creatinine clearance. DOAC = direct oral anticoagulant. NVAF = non-valvular atrial fibrillation. OD = once daily. P-gp = glycoprotein.

The summary of medicines characteristics^44–49^ and Figure 1 list standard dose parameters for UK-licensed OACs. The appropriateness of DOAC administration was assessed by the primary author and oversight provided by the GP clinical lead. The number, type, and acceptance of pharmacist-led interventions were collated during the post-intervention study period. Pharmacists undertaking reviews were qualified as independent prescribers and received an educational update on AF management, before interventions were undertaken in October 2018, via a presentation, including key differences between OAC choices, key differences on efficacy, and safety points.

### Data analysis

Data collection parameters were decided on with consultation and agreement between the researchers, GP clinical teams, and the ethical committee at the University of Huddersfield. Data were initially entered into a spreadsheet using Microsoft Excel 2019 and all analyses were performed using R (version 4.1.3). Results from descriptive analyses were reported as means or percentages. The Shapiro–Wilk test was used to test for normality of the dataset. Differences between patient groups were tested using the *t*-test or the Wilcoxon signed-rank test as appropriate for the continuous variables, expressed as percentages; a χ^2^ test was used for categorical variables, expressed as mean ± standard deviation (SD). A *P*-value <0.05 was considered statistically significant.

## Results

The combined population of the practices as of April 2019 was 139 236. In total, 616 patients were on the AF register representing a prevalence of 0.4%, with 76.3% (*n* = 470) of these on established OAC treatment. Baseline characteristics are outlined in Table 1.

**Table 1.** Baseline characteristics of DOAC and warfarin users (*N* = 470)

Characteristic^a^	DOACs, *n* = 298	VKA, *n* = 172
**Age, mean (SD)**	68.06 (9.19)	66.46 (9.66)
**Sex, male**	158 (53.0)	99 (57.6)
**Sex, female**	140 (47.0)	73 (42.4)
**CHA**_**2**_ **DS**_**2**_ **VASc score, mean (SD)**	3.99 (1.76)	3.92 (1.81)
**CHA_2_DS_2_VASc score**		
1	10 (3.4)	13 (7.6)
2	47 (15.8)	17 (9.9)
≥3	241 (80.9)	142 (82.6)
**HAS-BLED score, mean (SD)**	1.95 (0.81)	1.91 (0.79)
**HAS-BLED score**		
0	12 (4.0)	8 (4.7)
1	66 (22.1)	37 (21.5)
2	150 (50.3)	91 (52.9)
3	66 (22.1)	35 (20.3)
4	4 (1.3)	1 (0.6)
**Creatinine clearance, ml/min**		
<15	0 (0)	3 (1.7)
15–29	21 (7.0)	16 (9.3)
30–49	90 (30.2)	46 (26.7)
50–79	116 (38.9)	66 (38.4)
≥80	71 (23.8)	41 (23.8)
**Thromboembolic events**		
Pre-review stroke	23 (7.7)	13 (7.6)
Post-review stroke	1 (0.3)	0 (0)
Pre-review pulmonary embolism	0 (0)	1 (0.6)
**Hospital attendance**		
Accident and emergency	22 (7.4)	12 (7.0)
Admission	0 (0)	1 (0.6)
Readmission	0 (0)	0 (0)
**Prior bleeding episode**		
Total bleeding episodes	2 (0.7)	2 (1.2)
Gastrointestinal	1 (0.3)	0 (0)
Haematuria	0 (0)	1 (0.6)
Epistaxis	1 (0.3)	0 (0)
Rectal	0 (0)	1 (0.6)

^a^ *n* (%) unless stated otherwise. DOAC = direct oral anticoagulant. SD = standard deviation. VKA = vitamin K antagonist.

### DOAC initiation

The most prescribed OAC at baseline across the study population was rivaroxaban (43.8%) followed by warfarin (36.3%). DOAC prescribing increased from 48.4% in the pre-intervention group to 54.4% in the post-intervention group (*P* = 0.03) (Table 2). The largest increases were for edoxaban 60 mg tablets and apixaban 5 mg tablets.

**Table 2.** Change in DOAC prescribing pre- and post-intervention

Anticoagulants	Pre-intervention, ***n*** (%)	Post-intervention, ***n*** (%)
**DOAC**	**298^a^**	**335**
Rivaroxaban 20 mg	160 (53.7)	168 (50.1)
Rivaroxaban 15 mg	45 (15.1)	48 (14.3)
Rivaroxaban 10 mg	1 (0.3)	1 (0.3)
Apixaban 5 mg	51 (17.1)	61 (18.2)
Apixaban 2.5 mg	28 (9.4)	31 (9.3)
Edoxaban 60 mg	8 (2.7)	18 (5.4)
Edoxaban 30 mg	1 (0.3)	4 (1.2)
Dabigatran 150 mg	2 (0.7)	2 (0.6)
Dabigatran 110 mg	2 (0.7)	2 (0.6)

^a^ *P* = 0.03 versus ‘post-intervention’ group. DOAC = direct oral anticoagulant.

### Inappropriate DOAC prescribing

Inappropriate DOAC prescribing was present in 24.5% at pre-intervention. Pre-intervention compared with the post-intervention was related to underdosing (9.7% versus 1.0%, *P* = 0.09) or overdosing (14.8% versus 0.7%, *P* = 0.01), with inappropriate DOAC prescribing reducing to 1.7% post-intervention. The most common DOAC that was inappropriately prescribed was rivaroxaban (underdose and overdose) followed by apixaban (underdose) (Table 3).

**Table 3.** Number of patients with inappropriate DOAC prescribing by criterion

Criterion	Pre-intervention, *n* (%),*n* = 73	Post-intervention, *n* (%), *n* = 5
**Underdosed**	29 (39.7)^a^	3 (60.0)
Apixaban		
SCr, age, weight	20 (69.0)	1 (33.3)
Frequency	2 (6.9)	1 (33.3)
Rivaroxaban, CrCl	7 (24.1)	1 (33.3)
**Overdosed**	44 (60.3)^b^	2 (40.0)
Rivaroxaban, CrCl	43 (97.7)	1 (50.0)
Edoxaban, CrCl	1 (2.3)	1 (50.0)

^a^ *P* = 0.09 versus ‘post-intervention’ group. ^b^ *P* = 0.01 versus ‘post-intervention’ group. CrCl = estimated creatinine clearance. DOAC = direct oral anticoagulant. SCr = serum creatinine.

### Bleed risk factors

Mean HAS-BLED scores were calculated and there was a reduction from 3.00±0.65 versus 2.22±0.79 (*P*<0.01), pre- intervention to post-intervention. Modifiable bleed risk interventions resulted in 73.0% (*n* = 54) of patients having a reduction in their HAS-BLED score by at least one point, with one patient reducing by two points. Overall, 47 (63.5%) patients had a reduction in their HAS-BLED risk category from a higher bleed risk to a lower one (Table 4).

**Table 4.** HAS-BLED intervention outcomes (*N* = 74)

Outcomes	*n* (%)
Patients whose HAS-BLED score reduced by at least 1 point	54 (73.0)
Patients whose bleeding risk was reduced to a lesser HAS-BLED risk category	47 (63.5)
*High to moderate*	46 (62.2)
Deprescribe: antiplatelet therapy	4 (8.7)
Deprescribe: NSAID	3 (6.5)
SBP ≤140 mmHg	33 (71.7)
Alcohol reduction (patient reported)	6 (13.0)
*Moderate to low*	1 (1.4)
Deprescribe: antiplatelet therapy	1 (1.4)

NSAID = non-steroidal anti-inflammatory drug. SBP = systolic blood pressure.

In 27.0% (*n* = 20) of patients who received an intervention to modify the bleed risk, the HAS-BLED score did not decrease despite an intervention being applied. Specifically, the deprescribing of NSAIDs was not achieved in eight (40.0%) patients owing to patient preference, systolic blood pressure (SBP) remaining >160 mmHg after intensification in antihypertensive treatments in six (30.0%) patients, and patient-reported alcohol intake remaining >8 units per week after counselling and signposting to available community-based services in six (30.0%) patients (Table 5).

**Table 5.** Pharmacist interventions not resulting in lower HAS-BLED risk score (*N* = 74)

Interventions	*n* (%)
Pharmacist interventions not resulting in lower HAS-BLED scores	20 (27.0)
** Alcohol**	
Unit intakes remain >8 per week (patient reported)	6 (30.0)
** Deprescribing**	
NSAID: patient declined to stop	8 (40.0)
** Blood pressure**	
SBP remained >160 mmHg	6 (30.0)

NSAID = non-steroidal anti-inflammatory drug. SBP = systolic blood pressure.

### VKA to DOAC transition

In total, 36.6% of all patients on OAC were on VKA therapy, warfarin (*n* = 168) and acenocoumarol (*n* = 4), with a median duration of treatment of 4.05 years. TTR was available for 16.9% (*n* = 29) of patients on VKAs (median 92.5%, range 69–100). Table 6 shows their eligibility for conversion to a DOAC, with 25.7% of patients transitioning from VKA therapy to a DOAC, and summarises the reasons patients were unsuitable for transition to DOACs.

**Table 6.** Eligibility for transition from VKA therapy to a DOAC

Patient eligibility for conversion to a DOAC	*n* (%)
Patients on VKA as of 1 November 2018	172
Contraindication to DOACs	24 (14.0)
History of acute DVT or PE	1 (0.6)
Adverse drug reactions to DOACs	4 (2.3)
Medicine use causing drug interactions with DOACs	18 (10.5)
Named GP deemed not suitable for conversion	90 (52.3)
**Eligible patient conversion status (*n* = 35)**	
Successfully converted to a DOAC	9 (25.7)
Patients that could not be converted	26 (74.3)
**Reasons for non-conversion of eligible patients (*n* = 26)**	
Patient preference	18 (69.2)
Cost: FP10 NHS prescription charges	5 (19.2)
Unavailable for follow-up	3 (11.5)

DOAC = direct oral anticoagulant. DVT = deep vein thrombosis. PE = pulmonary embolism. VKA = vitamin K antagonist.

## Discussion

### Summary

This study demonstrated that pharmacists in general practice could improve the management of AF. There are three main findings in this study: 1) the overall prescribing of OAC increased by 6.0% in those patients with AF where OAC is currently not initiated; 2) optimising medicines, and in particular optimising DOAC dosing, in line with guidance and individual patient parameters, is potentially a valuable role for pharmacists in general practice, particularly overdose, as there was a reduction in overall inappropriate prescribing of DOAC by 11.0%; and 3) proactive management of modifiable bleeding risk factors by pharmacists in general practice could reduce the risk of harm commonly associated with DOAC use.

### Strengths and limitations

This study has successfully provided evidence that pharmacists in general practice can effectively improve the use of OAC therapies in patients with AF. Pharmacist-led interventions were successful in promoting higher prescription rates for DOACs in patients with AF. Furthermore, these interventions effectively optimised the dose of DOACs while considering bleeding risk. However, this study has some limitations and should be interpreted with caution. The study was conducted across 21 general practices, with varying processes, and differences in patient demographics, ethnic group, and deprivation were not considered. Therefore, the results may not be generalisable to other general practices in the UK. Furthermore, no economic analysis or evaluation was included, and patient follow-up was limited to the study period.

### Comparison with existing literature

The study population represented the entire AF register across the 21 general practices, with a prevalence of 0.4%. Although this study did not measure the effects of screening and AF identification, the study prevalence remains below the estimates of 1.1% AF prevalence for this region.^5^ This may be explained by the findings of the Bradford AF study, which concluded that people from a South Asian ethnic group have a significantly lower prevalence of AF when compared with people from a White ethnic group despite having a higher frequency of risk factors for the development of AF.^50^

There is overwhelming evidence that increased OAC rates in patients with AF lead to marked reduction in stroke prevalence, and the benefits of anticoagulating outweighs the associated risks.^51^ DOAC modelling data suggest a lower number needed to treat than warfarin,^52^ although these medications still need to be managed carefully to avoid harm. The results of this study indicate an increase in DOAC prescribing in line with other published literature of approximately 6.0%,^37–39^ demonstrating the role a pharmacist may undertake in the long-term condition management of AF.

Previous studies have reported the inappropriate dosing of DOACs in the range from 7.7%^53^–32%,^54^ with underdosing being more frequently reported.^53–55^ In the present study, overdosing (14.8%) was more frequent than underdosing (9.7%) in those already prescribed DOACs. This might be explained by the differing study population characteristics and the length of time patients were already established on DOACs. A dosing issue may not have been present at the time of initiation but subsequent changes in clinical characteristics may have resulted in dosing being inappropriate, such as a decline in estimated CrCl.

These findings align with harm reduction strategies by reducing incorrect DOAC prescribing in hospitals,^56^ and in primary care studies where problematic polypharmacy and antiplatelet monotherapy for AF were reduced.^38,39^ To the authors’ knowledge, no other studies have reported on pharmacist-led interventions across the full range of modifiable aspects of bleed risk reduction in primary care, uncontrolled SBP management, concurrent medication use predisposing to bleeding, and addressing alcohol intake.

Prescribing trend data have outlined the increase in the growth of DOACs in comparison with VKA therapy.^8^ This may be owing to an increase in guideline adherence^1,7^ and more recently the transition of VKA therapy to DOACs as part of the NHS England priority workstreams in response to COVID-19.^57^ Despite the higher acquisition cost of DOACs compared with VKAs, the decline in AF-related strokes has resulted in incremental savings at a national level.^58^ This study reported on the number of VKA to DOAC transitions and the reasons transitions were not implemented. The main reason identified was the GP with primary responsibility for the patient’s clinical care deemed the transition not suitable and could reflect therapeutic inertia, with GPs hesitant to modify the OAC initiation by another physician.

### Implications for research and practice

This study has suggested there is a useful role for pharmacists in general practice in the overall management of AF. It has shown that pharmacists are able to increase DOAC prescribing rates for already established AF, optimise DOAC to the patient’s clinical characteristics, and proactively manage bleeding risk.

The bleeding risk identification and management reported as part of this study may be limited as the bleeding risk stratification tool recommended by NICE in its guideline update recommended the use of ORBIT,^7^ whereas HAS-BLED was the recommended tool for bleeding risk stratification during the study period.^6^ Pharmacists in general practice have shown benefits to chronic disease management and the quality use of medicines,^59^ and improved adherence to guidelines for OAC treatment for AF in primary care,^60^ which this study’s findings support.

DOAC initiation was guideline-led and patient-centred, and inappropriate DOAC use was reviewed retrospectively. This may support a recommendation for patients with AF and on OAC to remain high priority for targeted structured medication review. Further research around patient perceptions and experiences of a pharmacist-led service would serve to enhance the outputs of this study. The long-term sustainability of this type of intervention programme may rely on balancing fidelity with adaptability^61^ to aid generalisability of the results and requires further research.
